# α-synuclein overexpression in the retina leads to vision impairment and degeneration of dopaminergic amacrine cells

**DOI:** 10.1038/s41598-020-66497-6

**Published:** 2020-06-15

**Authors:** Elena Marrocco, Alessia Indrieri, Federica Esposito, Valeria Tarallo, Anna Carboncino, Filomena Grazia Alvino, Sandro De Falco, Brunella Franco, Maria De Risi, Elvira De Leonibus

**Affiliations:** 1 Telethon Institute of Genetics and Medicine, Telethon Foundation, Pozzuoli, Naples Italy; 2 Institute for Genetic and Biomedical Research (IRGB), National Research Council (CNR), Milan, Italy; 3 Institute of Genetics and Biophysics “Adriano Buzzati-Traverso”, National Research Council (CNR), Naples, Italy; 4 PhD program in Behavioral Neuroscience, Sapienza University of Rome, Rome, Italy; 5 Medical Genetics, Department of Translational Medicine, University of Naples Federico II, Naples, Italy; 6 Institute of Biochemistry and Cellular Biology (IBBC), National Research Council (CNR), Monterotondo, Rome, Italy

**Keywords:** Cell death in the nervous system, Retina, Parkinson's disease

## Abstract

The presence of α-synuclein aggregates in the retina of Parkinson’s disease patients has been associated with vision impairment. In this study we sought to determine the effects of α-synuclein overexpression on the survival and function of dopaminergic amacrine cells (DACs) in the retina. Adult mice were intravitreally injected with an adeno-associated viral (AAV) vector to overexpress human wild-type α-synuclein in the inner retina. Before and after systemic injections of levodopa (L-DOPA), retinal responses and visual acuity-driven behavior were measured by electroretinography (ERG) and a water maze task, respectively. Amacrine cells and ganglion cells were counted at different time points after the injection. α-synuclein overexpression led to an early loss of DACs associated with a decrease of light-adapted ERG responses and visual acuity that could be rescued by systemic injections of L-DOPA. The data show that α-synuclein overexpression affects dopamine neurons in the retina. The approach provides a novel accessible method to model the underlying mechanisms implicated in the pathogenesis of synucleinopathies and for testing novel treatments.

## Introduction

The two hallmarks of Parkinson’s disease (PD) are the formation of α-synuclein (α-syn**)** inclusions into Lewy bodies (LBs)^1^ and the degeneration of the nigrostriatal dopaminergic system leading to motor and cognitive deficits. Experimental evidence in animal models established a causal link between α-syn overexpression or mutations and the degeneration of dopaminergic neurons. Indeed, recombinant adeno-associated viral vector (rAAV)-mediated overexpression of human wild-type (WT) α-syn in the midbrain of adult rodents leads to progressive loss of nigral dopaminergic neurons^2,3^. The clinical observations that PD patients also have visual symptoms such as reduced visual acuity, low contrast sensitivity, altered electroretinogram (ERG) and disturbed color vision^4–7^ have led to the retina being considered as a potential biomarker for PD.

α-syn aggregates have been identified in the retina of PD patients and in particular in the Ganglion Cell Layer (GCL), the Inner Plexiform Layer (IPL) and the Inner Nuclear Layer (INL)^8^. Transgenic mouse models overexpressing α-syn show an accumulation of the protein in specific retinal cells depending on the promoter used for the expression^9^. A transgenic mouse model overexpressing a fused α-syn-GFP under the Platelet Derived Endothelial Growth Factor (PDGFβ) promoter presented dot-like deposits in the INL and in the retinal ganglion cells (RGC) that increased over time^10^ and led to their degeneration. However, none of these studies explored the effects of synucleinopathy on dopaminergic amacrine cells (DACs) of the retina, which are located in the INL^11–13^.

Dopamine (DA) neurotransmission in the retina is regulated by a single subpopulation of amacrine cells (A18 cells)^14^ that synthesize and release the DA neurotransmitter^15,16^. DA plays a crucial role in the modulation of light adaptation. Light stimuli activate dopaminergic amacrine cells, triggering dopamine release^17,18^. Extracellular DA acts on dopaminergic receptors. In particular, D1-like receptors are expressed not only by amacrine cells but also in horizontal and bipolar cells while rods and cone mainly express D4R. D4R stimulation reduces the rod-cone communication through the gap junctions, which increases the direct response of cones in photopic conditions^14–16^. By contrast, the absence of DA favors the rod-cone conductance and shunting of the cone electrical signal. Retinal dopaminergic tone is thought to amplify the cone pathway producing a shift from rod-dominant to cone-dominant vision during daylight^19–21^. DACs also contribute to color vision. These visual functions are frequently compromised in Parkinsonian patients, although they respond positively to DA replacement therapy with levodopa (L-DOPA)^15,22^.

In this study we show, for the first time, that α-syn overexpression in the retina leads to neurodegeneration of dopaminergic amacrine cells, causing retinal-specific defects and consequent visual impairment.

## Materials and Methods

### Subjects

All the experiments were performed in male and female inbred C57BL/6 J adult (12–20 week-old) mice. Group housed mice, with *ad libitum* access to water and food, were maintained at 22 ± 1 °C and 55 ± 5% relative humidity, with a 12-h light: 12-h dark cycle (lights on 07:30–19:30) and tested during the light phase. The experiments were conducted in accordance with the European Communities Council directives and Italian laws on animal care. All experimental protocols were approved by the Italian Ministry of Health.

### Intravitreal injections

Animals were intravitreally injected with the same recombinant adeno-associated viral vector (rAAV) 2/6 expressing human (hu) α-synuclein (rAAV2/6-hu-α-syn) or GFP (rAAV2/6-GFP) under the control of the synapsin-1 promoter (7.7 × 10^13^ genome copies/ml) as previously described^2^. Intravitreal injection of rAAV2/6 has been previously reported to transduce the retina in mice and rats^23,24^. Pupils of anaesthetized animals (100 mg/kg methadomidine and 0.25 mg/kg ketamine) were dilated using 1% tropicamide and 2.5% phenylephrine (Chauvin, Essex, UK) and a small guide hole was made under the limbus with a 30 G needle. The eye was gently massaged with a cotton swab to remove a portion of the vitreous to avoid a post-injection reflux of vitreous and/or drug solution. Then, 1 µL of vector was intravitreally injected through the initial hole using a 34 G Hamilton syringe.

### 6-hydroxidopamine experiment

Two further groups of mice were intravitreally injected with the dopamine-specific neurotoxin 6-hydroxydopamine (6-OHDA) (2 µg/µL, Sigma Aldrich, 1 µL/side), which is classically used as a pharmacological model of dopamine cells neurodegeneration^25^. Intravitreal injections were performed as described for the viral vector injection (see above). 15–20 days after the injection procedure, mice underwent the behavioral procedure.

### Visual acuity test

We used a behavioral procedure of the visual acuity task modified from Prusky’s^26^ and Robison’s^27^ procedures.

#### Apparatus

Animals were tested in a circular tank (150 cm diameter, 35.5 cm high) filled with water (22 ± 1 °C). A rectangular white platform (37 cm long x 13 cm wide x 14 cm high) was submerged 1 cm below the water surface with a steel divider (46 cm long) extending toward the center of the pool that divided it into two equal quadrants; the divider constituted the response choice point. Two cards (40 × 44.5 cm) with vertical patterns of black and white stripes of different width were fixed to the wall of the pool in each quadrant. The escape platform was located in front of the card with smaller stripes. Animals discriminated between a card with larger stripes and a card with smaller stripes where the escape platform was located. Correct responses were recorded as direct entry in the quadrant where the card with smaller stripes was located. Visual acuity was finally converted in cycles/degree (c/d) as previously described in the literature^28^. To calculate visual acuity expressed in c/d, we have calculated visual acuity considering the distance from the maze divider (46 cm), the choice point; in line with previous studies^26,28^ maximal visual acuity in control mice was about 0.40 c/d.

#### Procedure

The task consisted of three phases: 1. During the shaping phase (day 1), animals were habituated to the task by positioning the card with 10 cm black and white stripes and the platform in the same quadrant to favor the association between the card and the submerged platform. The platform and the card were randomly positioned in the left or right quadrant for 3 sessions of 6 trials. Animals were released in the pool at progressively greater distance from the choice point in each session. 2. Training phase: the 10 cm black and white striped card and the 1 cm black and white striped card were randomly positioned in the left or the right quadrant and the platform was always located under the smaller striped card. Animals were trained to discriminate between two cards and to learn that the platform was always associated with the smaller striped card. They were tested for a maximum of 3 sessions per day (10 trials/session, 60 seconds/trial). If the animal made 70% correct responses (criterion) in a session or 4 consecutive correct responses, it was tested in the next phase; if the animals did not reach the criterion, they were re-tested in the training phase for a maximum of 3 days (3 sessions per day) 3. Test phase: couples of cards used were progressively more difficult to discriminate: 10 cm striped card vs. 1 cm striped card, 10 cm striped card vs. 2 cm striped card, 5 cm striped card vs. 2 cm striped card, 4 cm striped card vs. 3 cm striped card. To go to the next couple of cards mice were required to make 70% correct responses or 4 consecutive correct responses at the previous couple of cards. Animals were tested for a maximum of 3 sessions per day (10 trials/session, 60 seconds/trial).

#### L-DOPA treatment

After the visual water maze task was completed, animals were re-submitted to it, a week apart, to test the effects of systemic injections of L-DOPA or vehicle. Benserazide (20 mg/kg, Sigma Aldrich) in PBS 1x was injected intraperitoneally (i.p.) 15 minutes before L-DOPA (10 mg/kg, Sigma Aldrich, in saline solution, i.p.) or saline. Each animal received L-DOPA and saline 5–7 days apart in a randomized order.

#### Effects of L-DOPA on albino CD-1 outbred mice

To control for any non-specific effect of L-DOPA on motor performance, motivation or learning, we added a group of male and female outbred albino CD-1 mice (12–20 weeks old) (n = 10), which was tested in the visual acuity task and then treated with L-DOPA or vehicle.

### Electrophysiological recording

Mice were anesthetized with intraperitoneal injection of mixed ketamine/methadomidine (100 mg/kg and 0.25 mg/kg respectively) and accommodated in a stereotaxic apparatus; their pupils were dilated with 1% tropicamide and 2.5% phenylephrine (Chauvin, Essex, UK) and the body temperature was maintained at 37.5 °C. The electrophysiological signals were recorded through gold plate electrodes inserted under the lower eyelids. Electrodes in each eye were referred to a needle electrode inserted subcutaneously at the level of the corresponding frontal region. The different electrodes were connected to a signal conditioner (BNC 2089A- National Instruments) with a two-channel amplifier. Electrical signals were amplified (10000-fold) and band-pass filtered (0.3–100 Hz) for acquisition.

#### Dark-adapted ERG

Mice were dark-adapted for 3 hours. After anaesthesia and mydriasis, mice, under dim red light, were accommodated in a stereotaxic apparatus. After electrode positioning, ERGs were evoked by flashes of different light intensities ranging from −4 to +1 log cd.s/m^2^, in scotopic conditions. For each animal group, mean of a-wave and b-wave amplitude were plotted based on light intensity.

#### Light-adapted ERG

ERGs, registered in the presence of a constant background illumination set at 50 cd.s/m2, were evoked by 10 ms flashes of 10.0 cd.s/m2 elicited at different time-intervals (from 0 minute to 8 minute; to intervals 2 minute for each step). Flashes were generated through a Ganzfeld stimulator (CSO, Florence, Italy). The b-wave amplitude was calculated as the difference between maximal positive and negative amplitude peaks; a-wave amplitude was calculated as the maximal negative peak from baseline. For each animal group, mean of b-wave recorded over time was plotted as a function of time. The effects of treatment with L-DOPA or vehicle were tested one week later as described for the behavioural testing, with the only difference being that L-DOPA was administered 90 min before recordings.

### Immunofluorescence

For immunofluorescence on retinal sections, control (Not Injected) and rAAV-injected mice were euthanized at different time points post injection (one, two or three months). The eyes were removed and fixed in 4% paraformaldehyde fixative solution (PFA), cryoprotected in PBS sucrose, embedded in OCT (Optimal cutting temperature) and cut by a cryostat at 12 μm. Sections were processed for immunofluorescence as previously described^2,29^, using the following primary antibodies: TH (AB152 Merck Millipore), hu-α-syn (AB211 Santa Cruz Biotechnology), hu-α-syn phosphorylated at Ser129 (AB51253, Abcam), GAD65 (198102 Synaptic System), NeuN (AB104225 Abcam), RBPMS (15187-AP Proteintech), ChAT (AB144P Chemicon) diluted 1:400. DAPI was used as a nuclear counterstain. Images were taken using a fully motorized Leica DM5500. Quantification of cell number was performed as described^29^. GAD65-, TH- and ChAT-positive cells in the INL, and NeuN- and RBPMS-positive cells in the GCL, were manually counted on an average of 8 serial retinal sections in the areas surrounding the optic nerve.

Retina flat mounts were analysed as previously described^30^ with minor modifications. Mice were deeply anesthetized and perfused with 4% PFA. The eyes were removed and placed in PFA for 1 h. Whole retinas were isolated and washed three times in PBS 0.1% Tween 20, for 5 min each at room temperature (RT). Retinas were then placed in blocking solution [10% fetal bovine serum (FBS), 0.3% Triton in PBS] for 1 h and incubated with 1:500 anti-TH antibody (AB152 Merck Millipore) overnight at 4 °C. Retinas were then washed in PBS 0.1% Tween, placed in a 1:500 Alexa fluor 568 goat anti-rabbit IgG (Invitrogen) secondary antibody solution for 2 h at RT, and mounted on glass microscope slides.

Images were obtained using a Leica DM5500 scanner microscope. Scanned images of the entire retina were automatically joined together using LAS X software. Total TH-positive cells in each retina were automatically counted using ImageJ software.

### Statistical analysis

Electrophysiological data were analysed using a one-way repeated measure ANOVA (between variable: rAAV2/6-GFP and rAAV2/6-hu-α-syn; repeated measures: time for ERG ligh-adapted and luminance for a-wave and b-wave). After L-DOPA treatment, a two-way repeated measure ANOVA was used (between variable: rAAV2/6-GFP/rAAV2/6-hu-α-syn and Saline/L-DOPA; repeated measures: time). A Duncan *post-hoc* test was used when appropriate. Cell counting was analysed with a one-way ANOVA. A nonparametric test (Mann-Whitney) was used for behavioural data with cut-off performance. The statistical significance was set at p < 0.05.

## Results

### α-synuclein overexpression in the retina leads to vision impairment which can be rescued by L-DOPA administration

**Figure 1:**
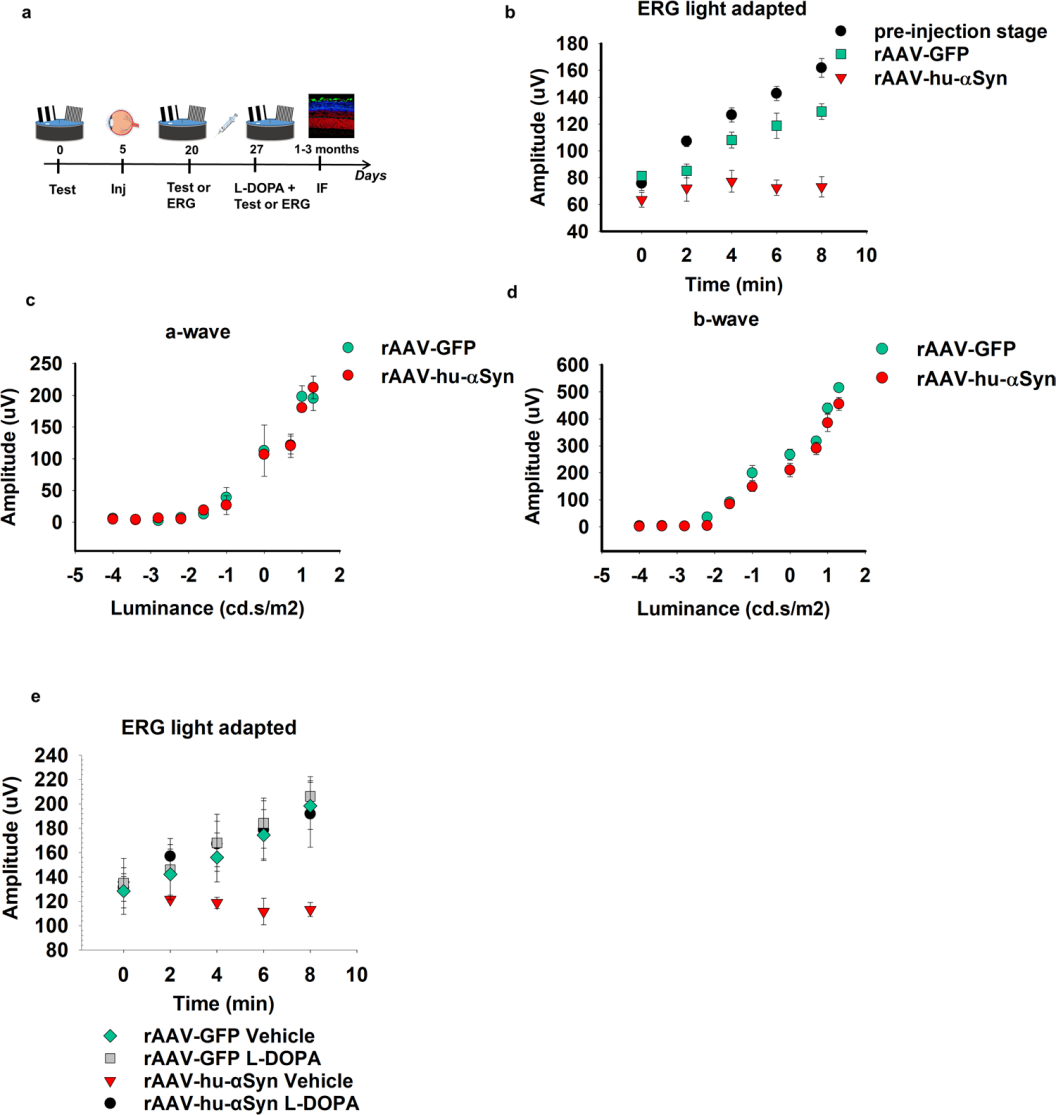
Elettroretinogram impairment in rAAV-hu-α-syn injected mice is rescued by L-DOPA. (**a**) Experimental design reporting the temporal order (days) of the experiments: the visual acuity test or ERG analysis before and after the rAAV injection (inj) and after L-DOPA or vehicle treatment followed by immunofluorescence (IF) analysis of the retina. (**b**) Light-adapted ERG measured over time (min), shows a severe and selective decrease of b-wave amplitude (mean) in rAAV-hu-α-syn injected mice compared to both rAAV-GFP mice and to the pre-injection time point [repeated measures ANOVA group F_(2,16)_=16.428; p = 0.0001; time F_(4,64)_=30.673; p < 0.0001, groups x time F_(8,64)_=11.473; p < 0.0001 (pre-injection, *n* = 10; rAAV-GFP, *n* = 4; rAAV-hu-α-syn *n* = 5), Duncan *post hoc* test]. ERGs were evoked by 10 ms flashes of 10.0 cd.s/m2 and registered in the presence of a constant background illumination (50 cd.s/m2). (**c-d**) No differences in a-wave and b-wave (mean) measured in the ERG dark-adapted condition were observed between rAAV-hu-α-syn and rAAV-GFP mice [**c:** repeated measure ANOVA group F_(1,9)_=0.018; p = 0.8951; luminance F_(9,81)_=88.406; p < 0.0001; group x luminance F_(9,81)_=0.321; p = 0.9659 (rAAV-GFP, *n* = 5; rAAV-hu-α-syn, *n* = 6); **d:** repeated measure ANOVA group F_(1,10)_=5.763; p = 0.0373; luminance F_(9,90)_=217.661; p < 0.0001; group x luminance F_(9,90)_=1.094; p = 0.3755 (rAAV-GFP, *n* = 5; rAAV-hu-α-syn, *n* = 8)]. **(e)** L-DOPA rescued the light-adapted ERG defect in rAAV-hu-α-syn [repeated measures ANOVA group x treatment x time F_(4,72)_=2.201; p = 0.0774 (rAAV-GFP Vehicle, *n* = 4; rAAV-GFP L-DOPA, *n* = 4; rAAV-hu-α-syn Vehicle, *n* = 4; rAAV-hu-α-syn L-DOPA, *n* = 10)]. ERGs were measured as in b.

α-syn was expressed in the retina of adult C57BL/6 J mice through an intravitreal bilateral injection of rAAV-hu-α-syn (experimental cohort) or rAAV-GFP (control cohort). We used a within-subject experimental design by testing the animal’s ability to adapt to light changes under rod saturating conditions with ERG and assessed their visual acuity in the water maze task, before and after rAAV injection (Fig. 1a). To assess the animals’ adaptation to light, a series of flashes were presented every 2 mins in the presence of a constant background. The stimulus- and time-dependent increase in b-wave amplitude (more detectable than the a-wave in rodents in photopic conditions^31^) observed in naïve mice was considered as an index of retinal response following light adaptation ERG analysis. GFP injection did not affect the pattern of increase, but it induced only a slight decrease in the amplitude of the response. rAAV-hu-α-syn induced a selective impairment in light-adapted responses (Fig. 1b) while those elicited in dark-adapted conditions were preserved (Fig. 1c,d, representative traces reported in Supplementary Fig. 1a). The amplitude defect of the b-wave in the light-adapted condition (Fig. 1b) was completely rescued by DA replacement *via* L-DOPA systemic administration (Fig. 1e, representative traces reported in Supplementary Fig. 1b). These ERG findings are similar to those performed in mice with retinal specific genetic ablation of tyrosine hydroxylase (TH), the key enzyme of the DA biosynthetic pathway^32^.

**Figure 2:**
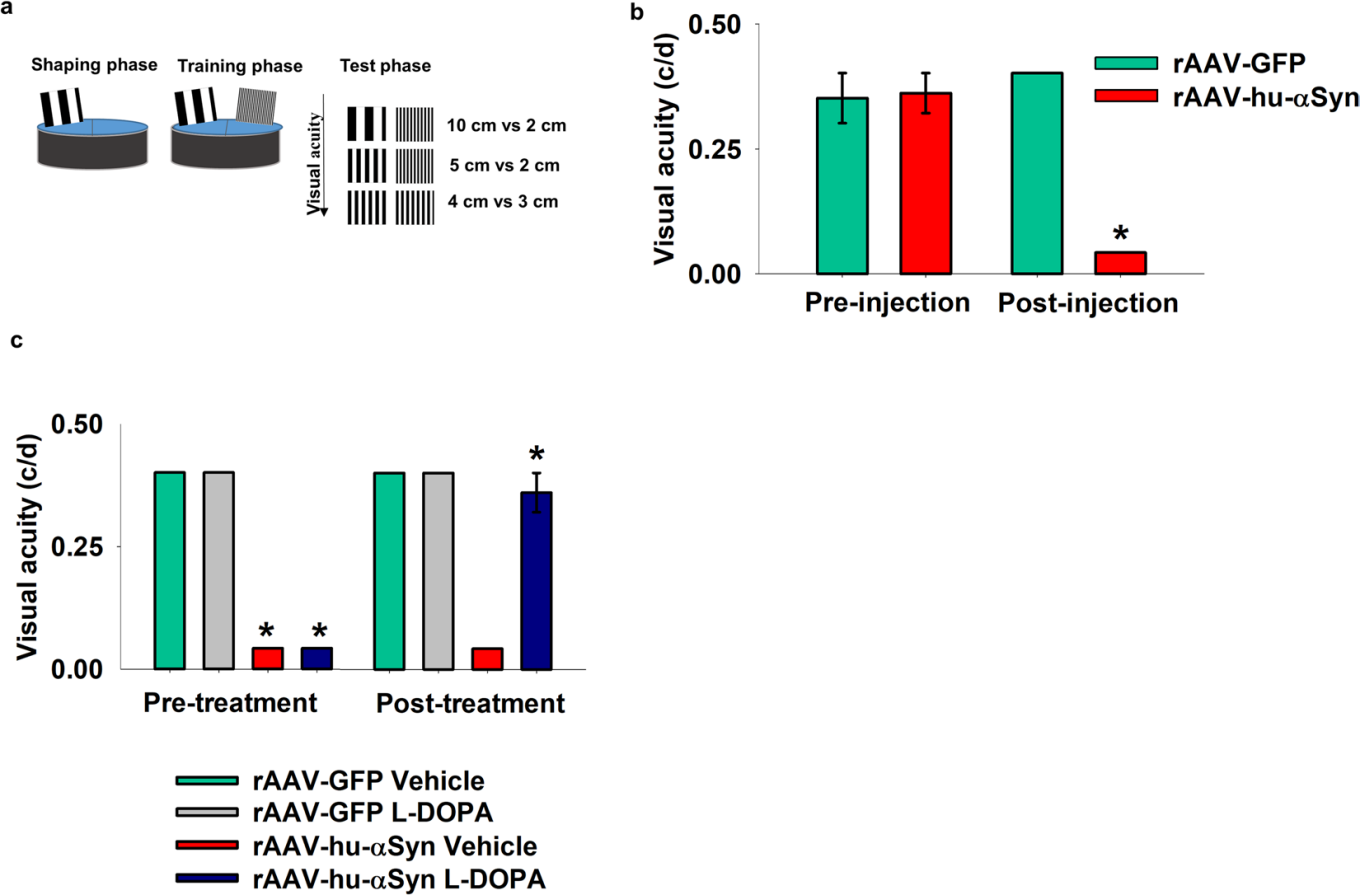
Visual acuity impairment in rAAV-hu-α-syn injected mice is rescued by L-DOPA. (**a**) Schematic representation of the different phases of the visual acuity test: 10 cm vs. 2 cm, 5 cm vs. 2 cm, 4 cm vs. 3 cm indicate the card couples used. (**b**) Impaired visual acuity (cycles per degrees, c/d) in rAAV-hu-α-syn injected mice compared to the pre-injection stage and rAAV-GFP mice [Pre-injection: Mann–Whitney U = 40, p > 0.99; Post-injection: Mann–Whitney U = 0, p = 0.0004 (rAAV-GFP, *n* = 4; rAAV-hu-α-syn, *n* = 5)]. (**c**) L-DOPA rescued the visual acuity defect in rAAV-hu-α-syn injected mice [Pre-treatment: AAV-injection Mann–Whitney U = 0, p = 0.0004, L-DOPA treatment: Mann–Whitney U = 40.5, p > 0.99; Post-treatment: AAV-injection Mann–Whitney U = 16, p = 0.03, L-DOPA treatment: Mann–Whitney U = 20, p = 0.07 (rAAV-GFP Vehicle, *n* = 4; rAAV-GFP L-DOPA, *n* = 4; rAAV-hu-α-syn Vehicle, *n* = 5; rAAV-hu-α-syn L-DOPA, *n* = 5)]. Some histograms lack error bars because there was no variability in the performance and all animals reached performance plateau. Data represent mean ± SEM. *p < 0.05 vs rAAV-GFP injection.

In order to evaluate whether α-syn overexpression in the retina leads to changes in visual acuity, we investigated the ability of α-syn injected mice to discriminate between two different spatial frequencies in a water maze task, under light conditions, similar to previously published behavioral procedures^28^ (Fig. 2a, see Materials and Methods). Untreated C57BL/6 J mice showed maximal visual acuity of about 0.40 c/d (Supplementary Video 1). rAAV-hu-α-syn injection significantly impaired visual acuity (0.04 c/d) compared to both the pre-injected and rAAV-GFP injected mice (Fig. 2b, Supplementary Video 2). A major advantage of this intravitreal selective expression of α-syn is that it allows to specifically relate the behavioral defects in the water maze with vision impairment, ruling out the role of motor impairment that is characteristic of genetic animal models of synucleinopathy. Interestingly, administration of L-DOPA rescued the visual acuity defect to pre-treatment conditions (Fig. 2c) (Supplementary Video 3).

**Figure 3:**
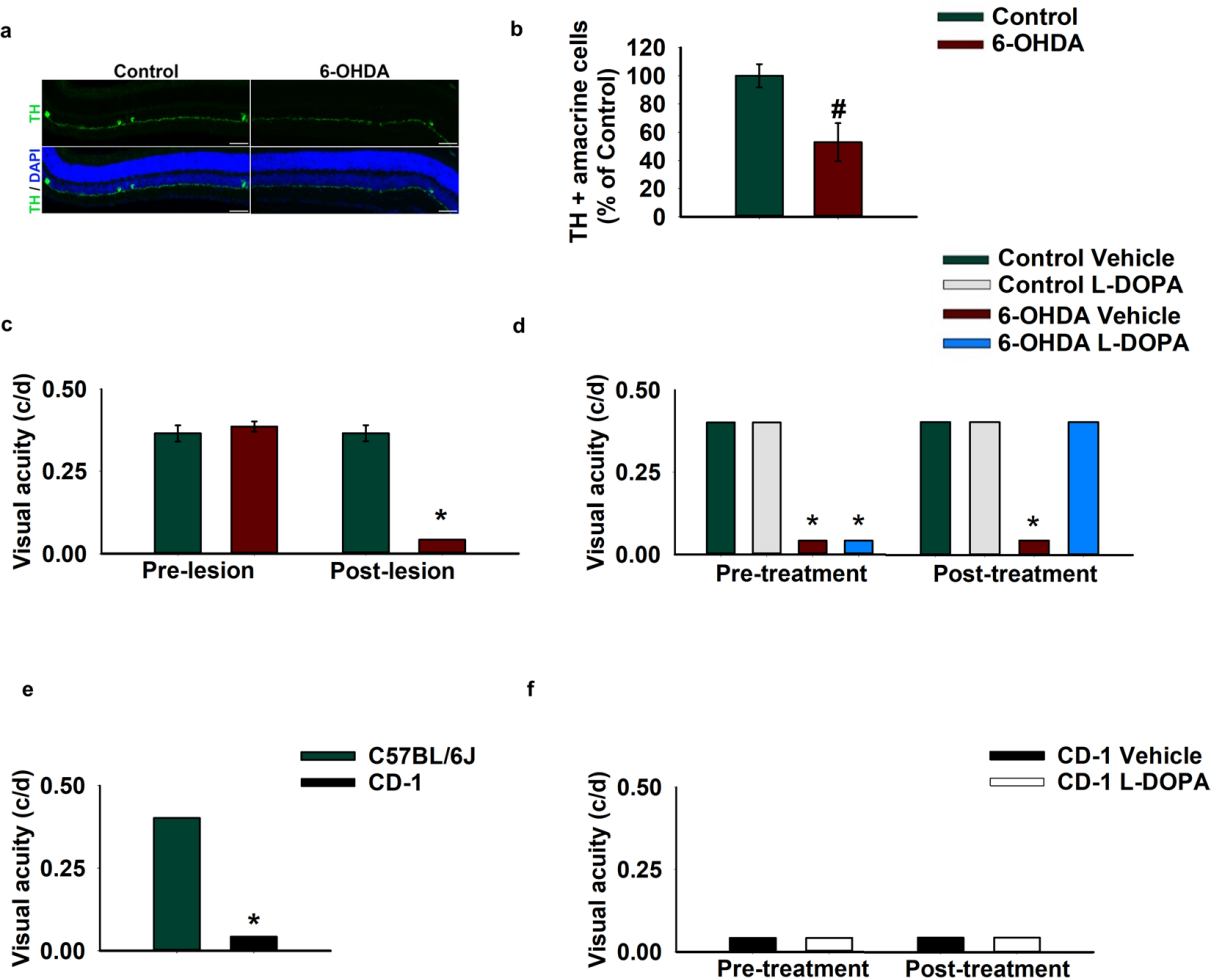
L-DOPA rescues the visual acuity impairment in 6-OHDA injected pigmented mice, but not in CD-1 albino mice. (**a**) Retinal sections showing TH positive (TH+) amacrine cells (green signal) in control and 6-OHDA injected mice (10X magnification, scale bar 50 µm). (**b**) Number of TH+ amacrine cells in control and 6-OHDA injected mice [one-way ANOVA group F_(1,9)_=8.687; p = 0.0163 (Control, *n* = 4; 6-OHDA, *n* = 7)]. (**c**) Visual acuity measured in cycles per degrees (c/d) in the pre-lesion and in the post-lesion stage. 6-OHDA caused a significant impairment in visual acuity [Pre-lesion: Mann–Whitney U = 13.5, p > 0.99, Post-lesion: Mann–Whitney U = 0, p = 0.0003 (Control *n* = 11; 6-OHDA *n* = 13)]. (**d**) L-DOPA rescues the visual deficit in 6-OHDA injected mice [Pre-treatment: 6-OHDA lesion Mann–Whitney U = 0, p = 0.0003, L-DOPA treatment Mann–Whitney U = 72, p > 0.99; Post-treatment: 6-OHDA lesion Mann–Whitney U = 27, p = 0.07, L-DOPA treatment Mann–Whitney U = 18, p = 0.0002 (Control vehicle *n* = 3; Control L-DOPA *n* = 3; 6-OHDA *n* = 9; 6-OHDA *n* = 9)]. Data represent mean ± SEM. # p < 0.05 vs Control; *p < 0.05 vs pre-lesion, pre-treatment stage. (**e**) Visual acuity measured in cycles per degr**e**es (c/d) in CD-1 and in C57BL/6 J mice [Strain: Mann–Whitney U = 0, p = 0.0002 (C57BL/6 J, *n* = 10; CD-1, *n* = 10)]. (**f**) L-DOPA or Vehicle treatment in CD-1 mice [Pre-treatment: Mann–Whitney U = 12, p > 0.99; Post-treatment: Mann–Whitney U = 12, p > 0.99 (Vehicle, *n* = 4; L-DOPA, *n* = 6)]. Some histograms lack error bars because there was no variability in the performance and all animals reached performance plateau. *p < 0.05 vs C57BL/6 J.

The rescue induced by L-DOPA in α-syn injected mice suggests that the visual defects induced by α-syn overexpression are specifically due to the loss of dopaminergic amacrine neurotransmission. Indeed, similar results were observed using a classical pharmacological model of DA neuronal loss that was obtained by injection of the DA selective neurotoxin 6-hydroxydopamine (6-OHDA). Intravitreal administration of 6-OHDA resulted in a significant ablation of the TH+ amacrine cells (Fig. 3a,b) leading to visual-acuity impairment (Fig. 3c), which was rescued by L-DOPA supplementation (Fig. 3d).

To further assess the specificity of the effects of L-DOPA on DACs, we tested CD-1 albino outbred mice, which have normal levels of DA and L-DOPA, but reduced melanin due to the lack of tyrosinase action on the melanin biosynthetic pathway^33,34^. As expected, albino mice showed reduced visual acuity relative to pigmented mice (Fig. 3e)^35^ and L-DOPA treatment was not sufficient to rescue the defects (Fig. 3f), in line with previous clinical findings showing similar negative results in albino patients^36^. The fact that L-DOPA did not improve performance in CD-1 outbred mice, as well as in rAAV-GFP mice, makes unlikely that the effects observed in rAAV-hu-α-syn were due to non-specific motor or cognitive improving effects of the drug.

### α-synuclein overexpression leads to time-dependent dopamine neuronal loss

**Figure 4:**
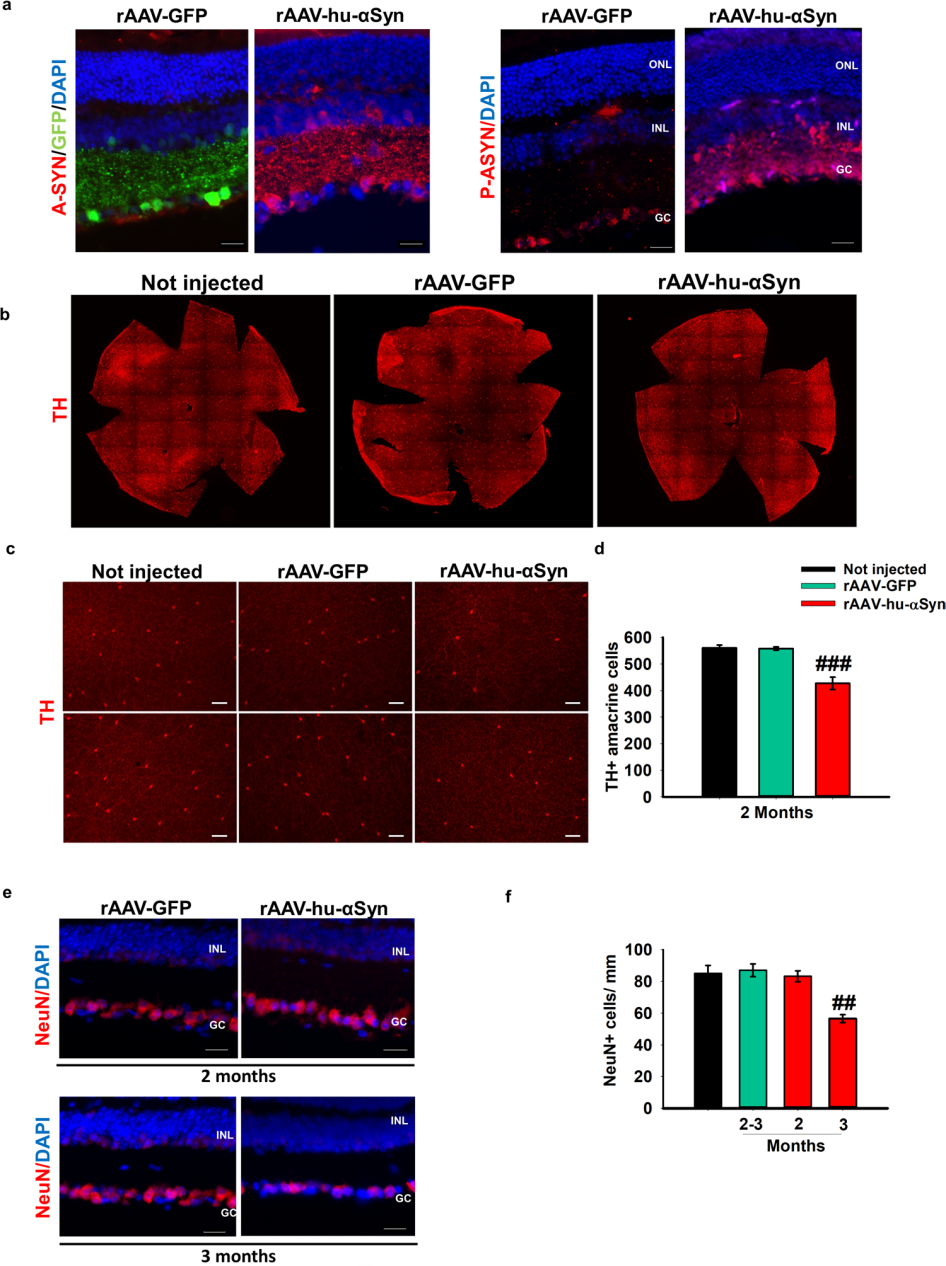
Reduction of TH+ amacrine cells precedes ganglion cell loss in rAAV-hu-α-syn injected mice. (**a**) Representative immunofluorescence showing retinal sections of rAAV-hu-α-syn injected mice and rAAV-GFP mice stained with antibody anti-hu-α-syn (A-SYN) (red) and antibody anti-phospho α-syn (P-ASYN) (red) (20x Magnification, Scale bar 50 µm). (**b**) Representative images of whole mount anti-TH immunofluorescence on Not injected, rAAV-GFP- and rAAV-hu-α-syn-injected retinas at 2 months post injection. (**c**) Representative micrographs from two quadrants of the retina flat mount of TH staining at 2 months post injection of rAAV-hu-α-syn compared to the controls (Not injected and retinas injected with rAAV-GFP) (10x Magnification, Scale bar 75 µm). (**d**) Total dopaminergic amacrine cells count on TH retina flat mount staining, at 2 months post injection of rAAV-hu-α-syn compared to the controls (Not injected and retinas injected with rAAV-GFP) [one-way ANOVA group F_(2,12)_=28.34; p < 0.0001 (Not injected, *n* = *6;* rAAV-GFP, *n* = *6;* rAAV-hu-α-syn, *n* = *6)*]. (**e**) Representative immunofluorescence showing retinal sections of rAAV-hu-α-syn injected mice (left) and rAAV-GFP mice (right) stained with antibody anti-NeuN (red) at 2 month and 3 months post injection (20x Magnification, Scale bar 50 µm) [one-way ANOVA group F_(3,7)_=9.173; p = 0.0080 (Not injected, *n* = *6;* rAAV-GFP, *n* = *4;* rAAV-hu-α-syn, *n* = *5)*]. Data represent mean ± SEM. ^##^ p < 0.01 vs rAAV-GFP; ^###^ p < 0.0001 vs rAAV-GFP.

To determine whether the underlying mechanisms of the functional findings correspond to specific histopathological effects, we analyzed the rAAV-injected retinas by immunofluorescence. One month after post-intravitreal administration, rAAV2/6-GFP efficiently transduced the inner retina including the IPL, the deepest INL and extensively the GCL (Fig. 4a, left panel). In addition, rAAV-hu-α-syn injected retinas were also positive for serine 129-phosphorylated α-syn (Fig. 4a, right panel), a specific biomarker of α-syn aggregates and/or misfolded protein^8,37^. In accordance with the functional data, we found a highly significant reduction of the number of TH-immunoreactive amacrine cells two months post-injection evaluated with whole mount slices (Fig. 4b–d) and in vertical retinal slices (Supplementary Fig. 2a,b), which further progresses at a later time point (Supplementary Fig. 2a,b). Ganglion cell numbers were not affected at two months after injection, as evaluated by NeuN staining. However, a significant reduction in the number of NeuN+ cells was detectable at a later time point (3 months) (Fig. 4e,f), as also confirmed by RBPMS staining that is selectively expressed in adult ganglion cells (Supplementary Fig. 2c,d). Notably, we did not find any change in the number of GABAergic glutamic acid decarboxylase 65 (GAD65)-positive amacrine cells and cholinergic amacrine cells expressing choline acetyltransferase (ChAT) (Supplementary Fig. 2e,f).

## Discussion

The observation of reduced dopamine innervation in the central retina of Parkinsonian patients associated with altered adaptation to light contrast and specific defects in visual acuity was made already 30 years ago^38^. We have confirmed previous findings obtained in genetic and pharmacological animal models, showing that loss of function of TH+ amacrine cells results in visual defects that can be corrected by L-DOPA replacement therapy^20,39,40^. More recent evidence has suggested that α-syn aggregates are present in the eyes of PD patients. Studies in transgenic animal models of α-synucleinopathy^9,10^ confirmed this finding and showed that phosphorylated α-syn accumulates in the retina in parallel with that in the brain, even in early stages preceding the development of clinical signs of parkinsonism or dementia^41^. However, synucleinopathy in the retina has never been directly associated with DACs neurodegeneration and consequent visual defects.

In this study, we report for the first time that α-syn overexpression in the retina induces a time-dependent loss of TH+ amacrine cells which precedes ganglion cell degeneration. In this model, no detectable changes in the number of GABAergic and cholinergic amacrine cells were observed. Many of the TH+ positive cells co-express GABA but TH+ are only a minor part of GABAergic amacrine cells in the INL. Therefore, the lack of significant changes in the number of GABA-positive amacrine cells might be explained by considering that DACs are only a minor fraction of the GABAergic cells^11–13^.

The loss of TH+ cells was associated with altered light-adaptation and impaired performance in the visual acuity version of the water maze task.

Although, the histological, electrophysiological and behavioral findings we provide in this study consistently suggest that the impairment in early stages was selectively induced by an impairment of DACs function, we cannot completely exclude that other neuronal populations might also be affected at this early stage. Nevertheless, the fact that systemic injection of L-DOPA, which selectively acts on DA neurons, completely rescued the visual defects strongly suggests that the early functional impairments induced by α-syn overexpression are selectively dependent on impaired DA cellular function. In Parkinsonian patients, L-DOPA rescues motor symptoms by favoring DA synthesis and replacement in the remaining dopaminergic cells (from 30 to 10% neurons) (for review see^42^). In our model only 50% of TH+ amacrine cells were destroyed so the rescuing effect of L-DOPA on visual function was likely modulated by its action on the remaining TH+ amacrine cells.

These findings show that α-syn exerts harmful effects on DA neurons independently from the cellular context in which they are integrated, including the retina.

The eye is gaining momentum as an object for studying neurodegenerative disorders due to its easy accessibility^43^. Interestingly, we could detect phosphorylated α-syn at this early stage in the retina, which has been suggested as a valid early marker of the pathology^41^. Indeed, it is being considered as an ideal model organ for both an early identification of protein aggregates and for testing novel therapeutic approaches in neurodegenerative disorders.

## Supplementary information

Supplementary Information.

Supplementary video 1.

Supplementary video 2.

Supplementary video 3.

## Data Availability

The data that support the findings of this study are available from the corresponding author upon reasonable request.
