# Supplementary material for: α-synuclein overexpression in the retina leads to vision impairment and degeneration of dopaminergic amacrine cells
Source: Sci Rep. 2020 Jun 15;10:9619. doi: 10.1038/s41598-020-66497-6 (PMC7295803; doi:10.1038/s41598-020-66497-6)
Supplement: Supplementary file 1 — Supplementary Information. [file 41598_2020_66497_MOESM1_ESM.pdf]

α-synuclein overexpression in the retina leads to vision impairment and degeneration of dopaminergic amacrine cells

Marrocco Elena*, Alessia Indrieri*, Esposito Federica*, Tarallo Valeria, Carboncino Anna, Alvino Filomena Grazia, De Falco Sandro, Franco Brunella, De Risi Maria, De Leonibus Elvira#.

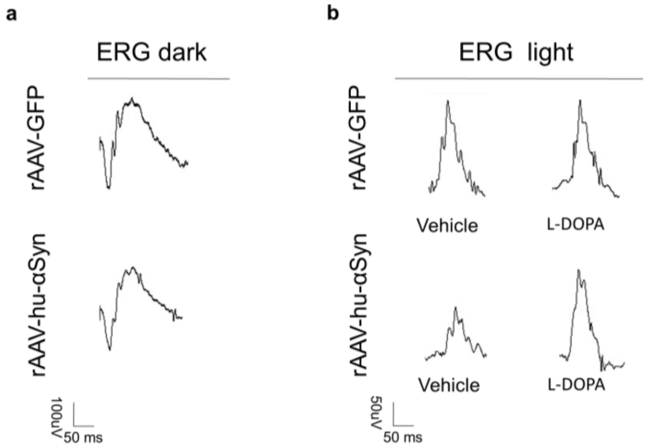

Supplementary Figure 1, related to Figure 1. (**a**) Representative ERG traces (in dark conditions) showing the maximum amplitude obtained with flashes of 10 cd.s/m2 in animals treated with rAAV2/6-hu-α-syn and rAAV2/6-GFP (control). (**b**) Representative ERG traces (light conditions) in animals treated with rAAV2/6-hu-α-syn and rAAV2/6-GFP (control) after treatment with vehicle or L-DOPA. The waves refer to retinal responses after 8 min of light adaptation (last point Figure 1e).

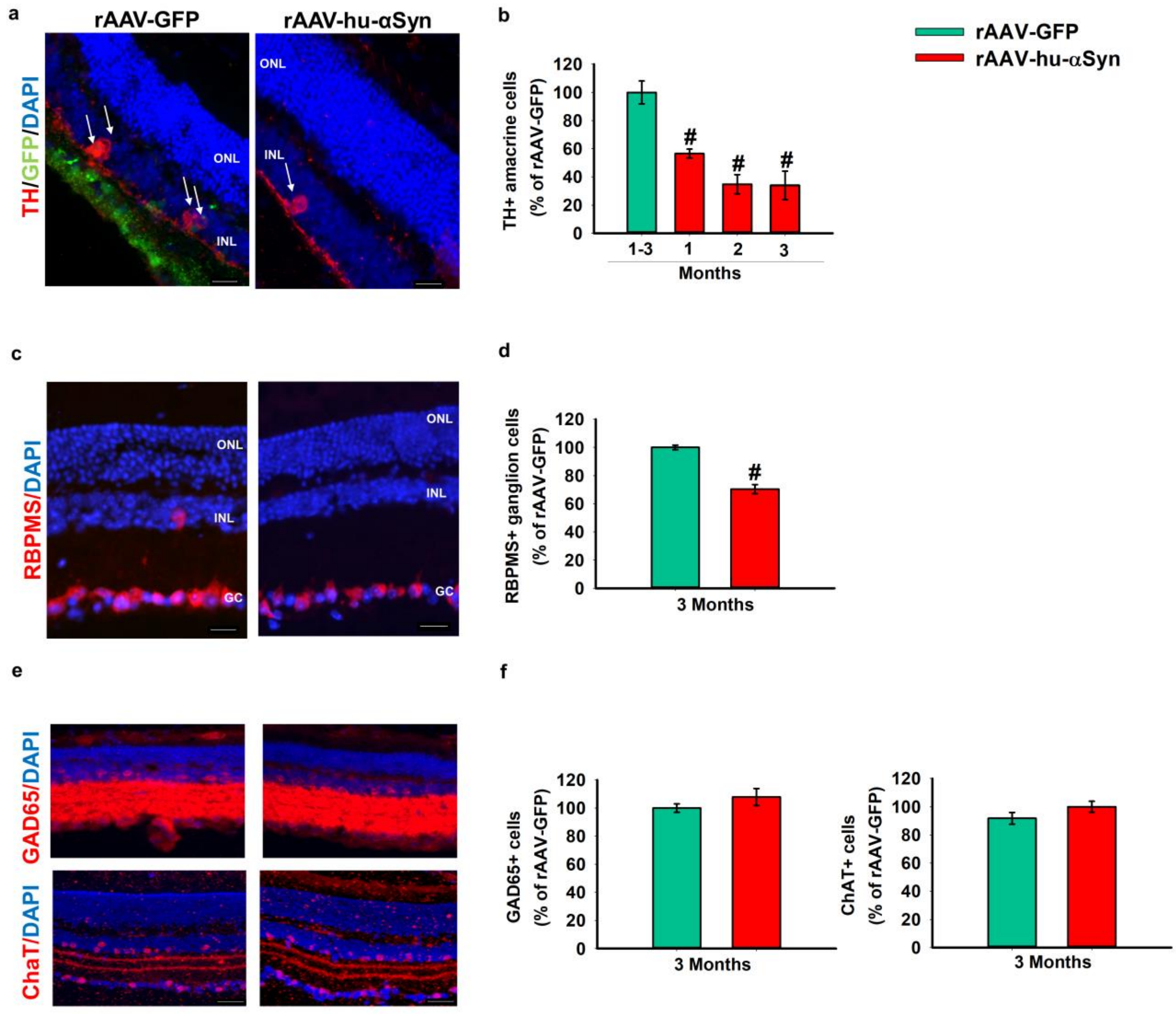

Supplementary Figure 2, related to Figure 4. Dopaminergic amacrine cells, but not GABAergic or cholinergic, are lost in α-syn injected retina. (**a-b**) Representative immunofluorescence on vertical retinal sections of rAAV-hu-α-syn injected mice (left) and rAAV-GFP mice (right) stained with antibody anti-tyrosine hydroxylase (TH) (red). White arrows indicate TH+ amacrine cells 1 month post-injection (10x Magnification, Scale bar 50µm), [one-way ANOVA group $F_{(1,3)}$=1161.600; p<0.0001]. (**c-d**) RBPMS staining (red) performed at 3 months post injection in rAAV-hu-α-syn injected mice and rAAV-GFP mice (right) (20x

Magnification, Scale bar 50μm) [one-way ANOVA, group $F_{(1,27)}=5.204$; $p=0.0306$]. (**e-f**) GAD65 and ChAT staining on retina of rAAV-hu-α-syn and rAAV-GFP- injected at 3 month post injection (20x Magnification, Scale bar 50μm) [GAD65: one-way ANOVA, group $F_{(1,41)}= 0.733$; $p=0.3970$; ChAT+: one-way ANOVA, group $F_{(1,28)}=2.128$; $p=0.155$]. Data represent mean ± SEM. # $p < 0.05$ vs rAAV-GFP.

Supplementary Videos. Videos showing visual task performance of C57BL/6J mice pre-injection (Video 1), after intravitreal injection of (rAAV-hu-α-syn) combined with saline (Video 2) and L-DOPA replacement therapy (Video 3). (**Video 1**) Representative sample trial of a C57BL/6J mouse during the visual task where it is required to discriminate between a 3 cm black and white striped card and a 4 cm black and white striped card. The platform is located under the 3 cm striped card. (**Video 2**) Representative trial of the visual task showing that bilateral rAAV-hu-α-syn intravitreal injection affects performance at the first stage of the procedure when animals are required to discriminate between a 1 cm black and white striped card and a 10 cm black and white striped card. The platform is located under the 1 cm striped card. (**Video 3**) Representative trial of the visual task showing that L-DOPA treatment rescues performance in rAAV-hu-α-syn injected mice on the same trial reported in Video 2.
